# Albumin-to-alkaline phosphatase ratio as a novel prognostic indicator in patients undergoing peritoneal dialysis: a propensity score matching analysis

**DOI:** 10.3389/fmed.2024.1302603

**Published:** 2024-04-18

**Authors:** Wenkai Xia, Xi Hua, Dong Sun, Xiangcheng Xie, Hong Hu

**Affiliations:** ^1^Department of Nephrology, Jiangyin People's Hospital Affiliated to Nantong University, Jiangyin, China; ^2^Nephrologisches Zentrum, Medizinische Klinik und Poliklinik IV, Klinikum der Universität München, Ludwig-Maximilians-University Munich, Munich, Germany; ^3^Department of Nephrology, Affiliated Hospital of Yangzhou University, Yangzhou First People's Hospital, Yangzhou, China; ^4^Department of Nephrology, The Affiliated Suzhou Hospital of Nanjing Medical University, Suzhou Municipal Hospital, Suzhou, China; ^5^Department of Nephrology, Affiliated Hangzhou First People's Hospital, Zhejiang University School of Medicine, Hangzhou, Zhejiang, China

**Keywords:** albumin, alkaline phosphatase, peritoneal dialysis, propensity score matching, prognosis

## Abstract

**Background:**

Though the albumin-to-alkaline phosphatase ratio (AAPR) is used as a biomarker in various diseases, little is known about its effect on outcomes after peritoneal dialysis (PD).

**Methods:**

This multicenter retrospective study comprised 357 incident PD patients stratified according to the AAPR. Propensity score matching (PSM) was performed to identify 85 patients for a well-matched comparison of all-cause and cardiovascular mortality. Using Cox regression, we performed univariate and multivariate analyses to investigate the prognostic value of the AAPR and established a Kaplan-Meier curve-predicted nomogram to estimate expected overall survival (OS). We assessed the predictive accuracy using the concordance index (c-index).

**Results:**

We found that the optimal cut-off of the AAPR to predict mortality was 0.36. In the present cohort of patients undergoing PD, a low AAPR strongly correlated with worse OS. In the multivariate analysis, the AAPR was shown to be an independent marker predicting reduced OS both before [hazard ratio (HR) 1.68, 95% confidence interval (CI) 1.08–2.60, *P* = 0.020] and after PSM (HR 1.96, 95% CI 1.06–3.62, *P* = 0.020). We also observed significant differences in OS in several subgroups, but not the group of patients with comorbidities. A nomogram was established to predict overall survival, with a c-index for prediction accuracy was 0.71 after PSM.

**Conclusion:**

AAPR has potential as an independent prognostic biomarker in patients undergoing PD.

## Introduction

Patients diagnosed with chronic kidney disease (CKD) have an exceptionally high health and economic burden. In the Kidney Disease Improving Global Outcomes (KDIGO) guidelines, serum albumin and alkaline phosphatase (ALP) are recommended as adjunctive tests to assess nutrition status and bone turnover. Previous studies suggested that derangements in these indicators are associated with mortality in both patients with non-dialysis-dependent CKD and dialysis patients (1–5).

ALP was previously proposed as a biomarker for mineral and bone disorders in patients with CKD (6). In the general population, elevated serum ALP is associated with a higher risk of cardiovascular disease (CVD) and all-cause mortality, as it is in both non-dialysis CKD patients and prevalent dialysis patients [both peritoneal dialysis (PD) and hemodialysis (HD)], potentially through the progression of vascular calcification (4, 7). Thus, ALP is not only a biomarker of bone metabolism, but may be a predictive indicator of mortality in healthy individuals and patients with increased risk of CKD. Albumin levels reflect the status of inflammation, as well as malnutrition. Low serum albumin is recognized as a feature of protein-energy wasting and has been associated with increased mortality in dialysis patients (8, 9). Recently, a novel prognostic index based on albumin and ALP levels, the albumin-to-ALP ratio (AAPR), was proposed in a variety of cancers (10–12). However, the relationship between the AAPR and prognosis in patients undergoing PD has not yet been investigated.

The present study evaluated the association of AAPR with all-cause and CVD mortality in patients undergoing PD. Prognostic nomograms were established to better predict clinical outcomes in incident PD patients.

## Materials and methods

### Patients and study design

This study was a retrospective, multi-center, observational cohort study that included medical records from 357 incident PD patients at Jiangyin People's Hospital Affiliated to Nantong University, Affiliated Hospital of Yangzhou University, Yangzhou First People's Hospital, Affiliated Hangzhou First People's Hospital, Zhejiang University School of Medicine, and Affiliated Suzhou Hospital of Nanjing Medical University, Suzhou Municipal Hospital between January 2011 and December 2020. Individuals were excluded from the analysis if they were aged <18 years, had a history of HD or renal transplantation, had fewer than 3 consecutive months of PD, had been catheterized in other hospitals, or lacked baseline AAPR. Each patient was followed until death or transfer to HD or renal transplantation, or censored December 2020.

### Clinical and laboratory measurements

Clinical and laboratory data were collected within 3 months of the patient starting PD. The demographic data obtained for the present study were age, gender, body mass index (BMI), and major comorbidities [history of diabetes mellitus (DM), hypertension, and CVD]. Clinical biochemical data comprised systolic blood pressure (SBP), diastolic blood pressure (DBP), hemoglobin, blood urea nitrogen (BUN), creatinine, ALP, albumin, intact parathyroid hormone (iPTH), calcium (Ca), phosphorus (P), renal *Kt/v*, total *Kt/v*, and estimated glomerular filtration rate (eGFR). AAPR was defined by dividng serum albumin concentration (g/l) by the ALP (U/l).

### Study definition

The primary endpoint was all-cause mortality, which was defined as the time from the PD treatment to death from any cause. The secondary endpoint was CVD-related mortality, which was considered to be death caused by acute myocardial infarction, cardiac arrhythmia, congestive heart failure, cardiomyopathy, atherosclerotic heart disease, cerebrovascular accident, anoxic encephalopathy, or peripheral vascular disease. Patients with a history of type 1 or 2 DM or who were receiving current therapy with oral hypoglycemic agents or insulin were considered as having DM. Patients who reported current use of antihypertensive drugs or had two separate blood pressure measurements ≥140/90 mmHg were diagnosed with hypertension.

### Statistical analysis

Categorical variables are presented as frequencies and percentages and continuous variables as mean ± standard deviation. The categorical variables were compared using the Pearson χ^2^-test and continuous variables using the Mann-Whitney *U* or Kruskal-Wallis test. Survival rates were evaluated by Kaplan-Meier curves and log-rank tests. We performed Cox proportional hazards regression in univariate analyses and included the significant predictors in the multivariable analysis. To reduce bias, we performed propensity score matching (PSM) using age, gender, BMI, hypertension, DM, CVD, SBP, DBP, hemoglobin, BUN, creatinine, iPTH, Ca, P, *Kt/v*, and eGFR. Albumin and ALP were not included due to the confounders not being influenced by the AAPR in the PSM analysis. Furthermore, a nomogram and calibration curve were established using package *rms* in R 3.0.3. We performed all statistical analyses in SPSS 21.0 (SPSS Inc., IBM, USA) and R (version 3.2.2, Institute for Statistics and Mathematics, Vienna, Austria).

### Ethics approval and consent to participate

The study was approved by the Medical Ethics Committee of Jiangyin People's Hospital Affiliated to Nantong University, Affiliated Hospital of Yangzhou University, Yangzhou First People's Hospital, Affiliated Hangzhou First People's Hospital, Zhejiang University School of Medicine and Suzhou Municipal Hospital, it was conducted in accordance with the guidelines and regulations of the Helsinki Declaration. The need for informed consent was waived by the Institutional Review Board of Jiangyin People's Hospital Affiliated to Nantong University (IRB number 2021-0220), Affiliated Hospital of Yangzhou University, Yangzhou First People's Hospital (IRB number #376), Affiliated Hangzhou First People's Hospital, Zhejiang University School of Medicine (IRB number #1780), and Suzhou Municipal Hospital (IRB number 20-1206).

## Results

### Patient characteristics

**Figure 1:**
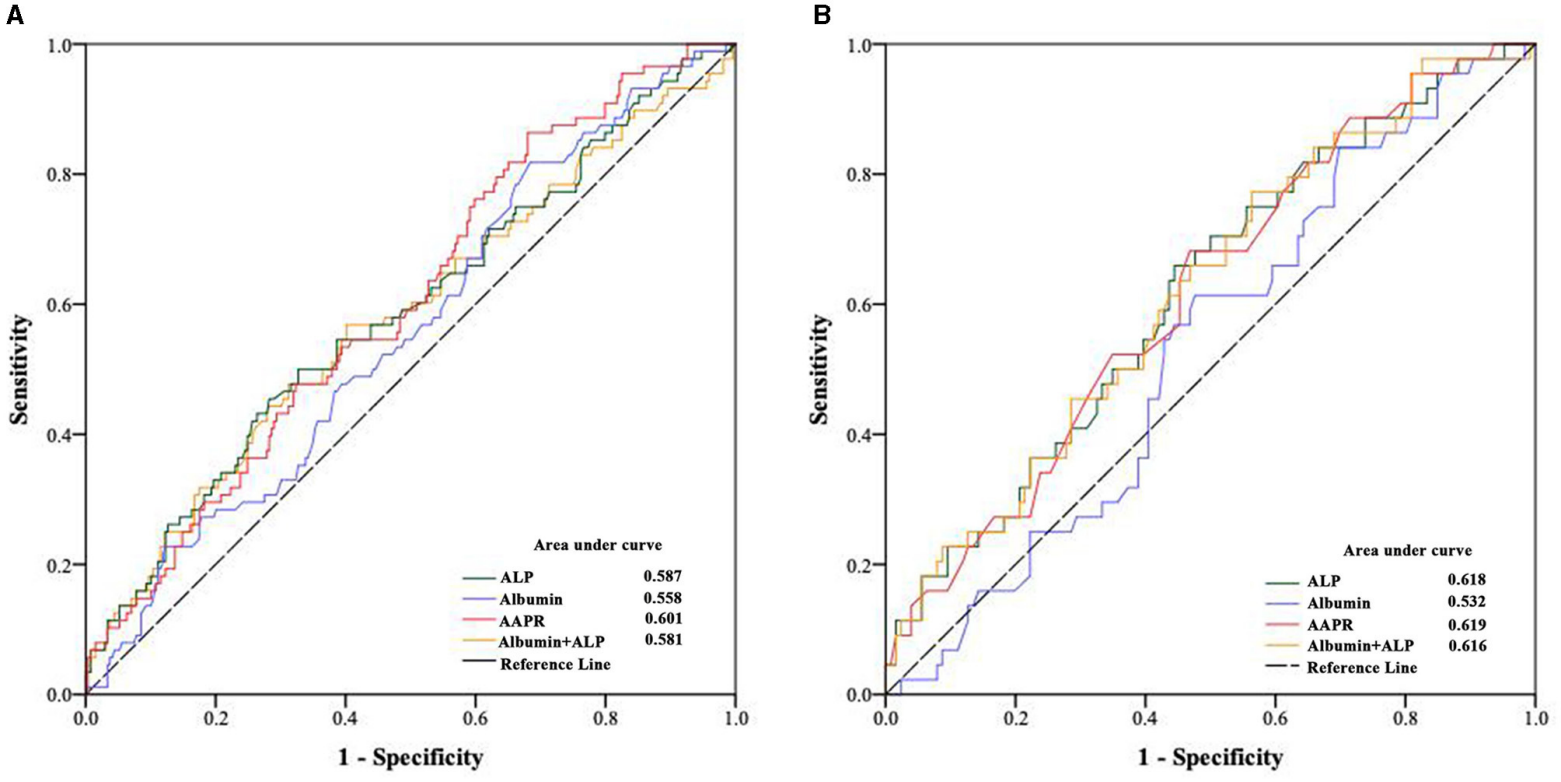
Optimal cutoff value for albumin, alkaline phosphatase and their combination for **(A)** all-cause and **(B)** cardiovascular disease mortality in peritoneal dialysis patients.

A total of 357 patients undergoing PD treatment were enrolled in this study. Compared to non-suvivors, surviors had a higher AAPR levels (Table 1). The optimal cutoff AAPR cut-off levels based on overall survival and cardiac-specific surval using receiver operating curve (ROC) analysis were determined to be 0.36 (Figure 1). We then divided the PD patients into two groups according to the cut-off threshold for subsequent analysis. The clinicopathological features of the enrolled patients undergoing PD according to their AAPR (low vs. high) before and after PSMare summarized in Table 2. Briefly, patients with lower AAPR levels were more likely to be older, with a history of DM, as well as lower DBP, albumin, creatinine, renal *Kt/v* and total *Kt/v*, and higher ALP and iPTH.

**Table 1:** Comparison of survivors and non-survivors.

	**Survivors (*n* = 269)**	**Non-survivors (*n* = 88)**	***P***
Age (y)	50 ± 16	57 ± 16	< 0.001
Gender (male/female)	156/113	54/34	0.577
BMI (kg/m^2^)	22.6 ± 3.5	23.6 ± 3.2	0.033
SBP (mmHg)	150.1 ± 23.2	151.3 ± 26.4	0.691
DBP (mmHg)	87.9 ± 16.3	86.1 ± 17.2	0.367
Smoking (*n*, %)	40 (14.9)	28 (31.8)	< 0.001
CVD (*n*, %)	17 (6.3)	17 (19.3)	< 0.001
DM (*n*, %)	58 (21.6)	31 (35.2)	0.010
Hypertension (*n*, %)	182 (67.7)	70 (79.5)	0.034
Hemoglobin (g/dl)	89.6 ± 21.5	92.3 ± 19.1	0.302
Albumin (g/l)	3.4 ± 0.5	3.3 ± 0.5	0.116
BUN (mmol/l)	23.3 ± 9.9	18.8 ± 7.7	< 0.001
Creatinine (umol/l)	834.6 ± 358.9	792.0 ± 329.0	0.325
ALP (U/l)	78.8 ± 36.8	102.9 ± 98.7	0.001
iPTH (pmol/l)	136.4 (24.8, 305.8)	26.7 (11.8, 92.3)	< 0.001
Ca (mmol/l)	2.1 ± 0.3	2.1 ± 0.5	0.190
P (mmol/l)	1.8 ± 0.6	1.7 ± 0.7	0.239
Total *Kt/v*	2.2 ± 0.7	2.3 ± 1.1	0.135
Renal *Kt/v*	1.1 ± 0.3	0.9 ± 0.2	0.412
eGFR (ml/min/1.73 m^2^)	6.3 ± 2.8	6.5 ± 2.9	0.739
AAPR	0.51 ± 0.22	0.43 ± 0.18	0.002
Observation period (m)	56 ± 12	28 ± 21	< 0.001

BMI, body mass index; SBP, systemic blood pressure; DBP, diastolic blood pressure; CVD, cardiovascular disease; DM, diabetes mellitus; BUN, blood urea nitrogen; ALP, alkaline phosphatase; iPTH, intact parathyroid hormone; Ca, calcium; P, phosphorus; eGFR, estimated glomerular filtration rate; AAPR, albumin to ALP ratio.

**Table 2:** Patient characteristics at baseline before and after PSM.

**Variables**	**Before matching**			**After matching**		
	**AAPR** ≤ **0.36 (*****n*** = **105)**	**AAPR** > **0.36 (*****n*** = **252)**	***P***	**AAPR** ≤ **0.36 (*****n*** = **85)**	**AAPR** > **0.36 (*****n*** = **85)**	***P***
Age (y)	55 ± 15	50 ± 16	0.014	55 ± 14	55 ± 16	0.922
Gender (male/female)	55/50	155/97	0.110	46/39	47/37	0.758
BMI (kg/m^2^)	23.2 ± 3.5	22.6 ± 3.5	0.156	23.3 ± 3.5	22.6 ± 3.7	0.241
SBP (mmHg)	151.6 ± 25.9	149.9 ± 23.2	0.529	150.1 ± 26.3	148.9 ± 23.5	0.745
DBP (mmHg)	83.6 ± 16.0	89.1 ± 16.5	0.004	83.8 ± 16.4	83.7 ± 13.9	0.964
Smoking (*n*,%)	21 (20.0)	47 (18.6)	0.767	13 (15.3)	18 (21.2)	0.517
CVD (*n*, %)	10 (9.5)	24 (9.5)	1.000	8 (9.4)	10 (11.8)	0.618
DM (*n*, %)	40 (38.1)	49 (19.4)	< 0.001	29 (34.1)	27 (31.8)	0.744
Hypertension (*n*, %)	78 (74.3)	174 (69.0)	0.322	64 (75.3)	65 (76.5)	0.858
Hemoglobin (g/dl)	8.8 ± 1.7	9.1 ± 2.2	0.125	8.8 ± 1.7	8.7 ± 2.0	0.650
Albumin (g/l)	32.6 ± 5.5	34.7 ± 4.6	< 0.001	33.1 ± 4.5	34.8 ± 4.7	0.017
BUN (mmol/l)	22.7 ± 10.6	22.0 ± 9.1	0.537	22.9 ± 10.5	22.5 ± 9.1	0.790
Creatinine (umol/l)	763.6 ± 316.4	849.3 ± 363.2	0.036	753.8 ± 299.6	747.4 ± 271.7	0.885
ALP (U/l)	135.0 ± 87.9	63.8 ± 16.8	< 0.001	137.5 ± 93.9	65.2 ± 17.0	< 0.001
iPTH (pmol/l)	168.9 (26.7, 379.2)	68.5 (16.9, 222.6)	0.005	189.8 (29.6, 442.5)	178.7 (26.1, 410.7)	0.715
Ca (mmol/l)	2.0 ± 0.3	2.1 ± 0.3	0.061	2.0 ± 0.3	2.1 ± 0.4	0.092
P (mmol/l)	1.7 ± 0.7	1.8 ± 0.6	0.337	1.7 ± 0.7	1.7 ± 0.5	0.908
Total *Kt/v*	2.0 ± 0.6	2.3 ± 0.9	0.011	2.0 ± 0.6	2.0 ± 0.6	0.422
Renal *Kt/v*	0.7 ± 0.3	1.1 ± 0.4	0.021	0.7 ± 0.3	0.7 ± 0.4	0.358
eGFR (ml/min/1.73 m^2^)	6.7 ± 3.0	6.3 ± 2.8	0.212	6.6 ± 2.8	6.7 ± 2.7	0.904

BMI, body mass index; SBP, systemic blood pressure; DBP, diastolic blood pressure; CVD, cardiovascular disease; DM, diabetes mellitus; BUN, blood urea nitrogen; ALP, alkaline phosphatase; iPTH, intact parathyroid hormone; Ca, calcium; P, phosphorus; eGFR, estimated glomerular filtration rate; AAPR, albumin to ALP ratio.

After PSM, 85 patients with a low AAPR (i.e., ≤0.36) were matched to 85 patients with a high AAPR (i.e., >0.36). The two groups were similar at baseline in regard to clinical and biochemical characteristics (Table 2).

### Association of the AAPR with survival outcomes

**Figure 2:**
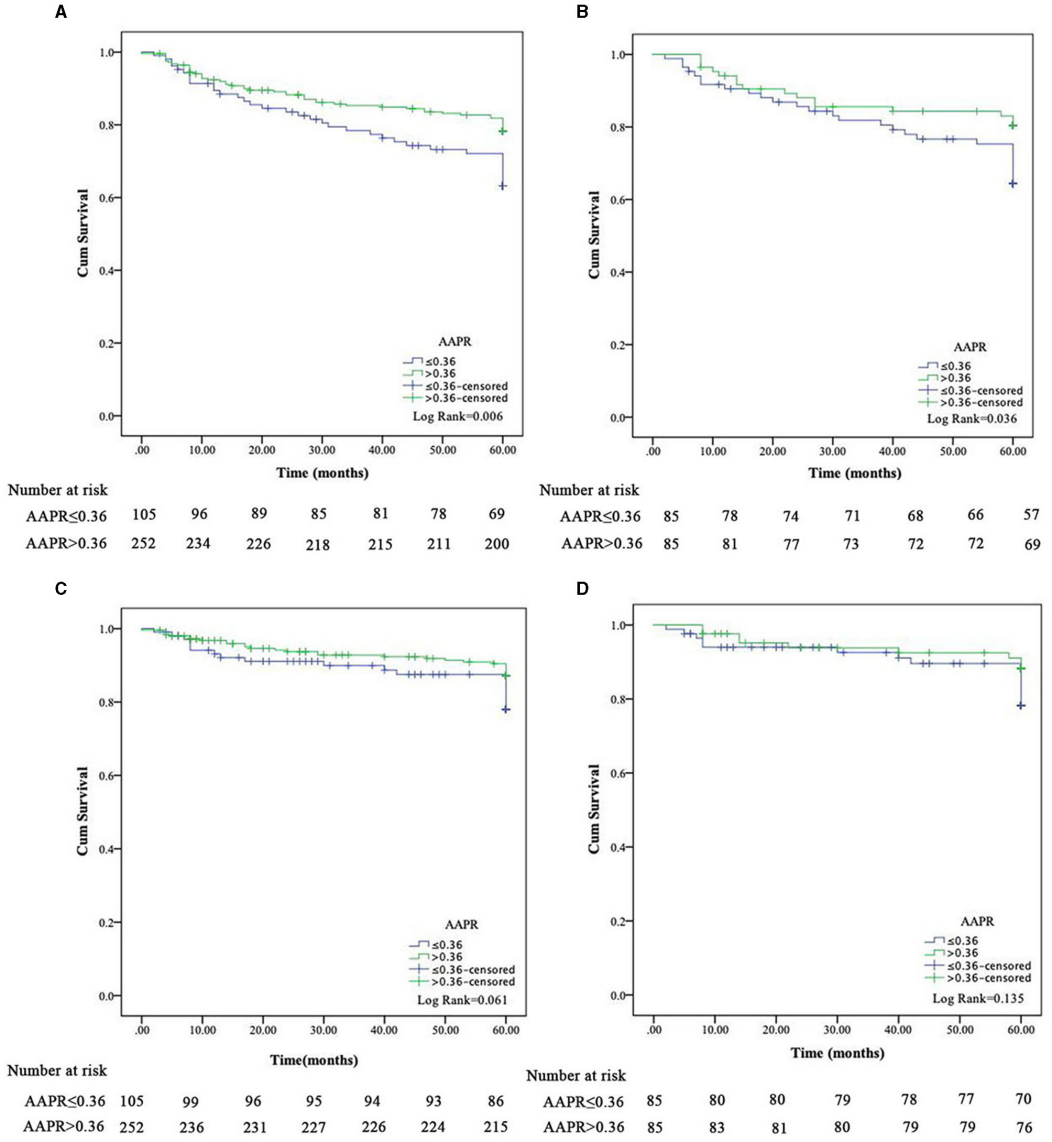
Kaplan-Meier survival curves for all-cause and cardiovascular disease mortality in peritoneal dialysis patients based on AAPR before **(A, C)** and after **(B, D)** propensity score matching.

In the Kaplan-Meier survival analysis and log-rank tests, AAPR ≤ 0.36 positively correlated with decreased overall survival (OS; Figure 2A, *P* < 0.05). Results from the multivariate Cox regression analysis revealed that a lower AAPR was independently associated with reduced OS [hazard ratio (HR) 1.68, 95% confidence interval (CI) 1.08–2.60, *P* = 0.020; Table 3]. However, the AAPR was not a significant prognostic factor in CVD mortality (HR 0.57, 95% CI 0.27–1.18, *P* = 0.129; Figure 2C and Table 4).

**Table 3:** Cox regression analysis of all-cause mortality before and after PSM.

**Variables**	**Before matching**			**After matching**		
	**Univariate analysis**	**Multivariate analysis**		**Univariate analysis**	**Multivariate analysis**	
	***P***	**HR (95% CI)**	***P***	***P***	**HR (95% CI)**	***P***
Age	< 0.001	1.02 (1.00–1.03)	0.022	0.244		
Gender (male)	0.518			0.651		
BMI	0.054			0.305		
SBP	0.743			0.864		
DBP	0.309			0.539		
Smoking	0.417			0.262		
CVD	< 0.001	2.06 (1.14–3.73)	0.016	0.093		
DM	0.011	1.26 (0.78–2.02)	0.348	0.043	1.87 (1.03–3.40)	0.039
Hypertension	0.040	1.34 (0.75–2.40)	0.330	0.533		
Hemoglobin	0.311			0.031	1.01 (0.99–1.03)	0.140
BUN	< 0.001	0.95 (0.92–0.97)	< 0.001	0.001	0.94 (0.90–0.98)	0.002
Creatinine	0.256			0.494		
iPTH	< 0.001	1.01 (0.99–1.03)	0.131	0.001	1.00 (0.98–1.02)	0.898
Ca	0.138			0.029	2.42 (0.84–5.95)	0.101
P	0.207			0.247		
Total *Kt/v*	0.166			0.051		
Renal *Kt/v*	0.216			0.117		
eGFR	0.551			0.619		
AAPR	< 0.001	1.68 (1.08–2.60)	0.020	0.041	1.96 (1.06–3.62)	0.032

BMI, body mass index; SBP, systemic blood pressure; DBP, diastolic blood pressure; CVD, cardiovascular disease; DM, diabetes mellitus; BUN, blood urea nitrogen; ALP, alkaline phosphatase; iPTH, intact parathyroid hormone; Ca, calcium; P, phosphorus; eGFR, estimated glomerular filtration rate; AAPR, albumin to ALP ratio.

**Table 4:** Cox regression analysis of cardiovascular mortality before and after PSM.

**Variables**	**Before matching**			**After matching**		
	**Univariate analysis**	**Multivariate analysis**		**Univariate analysis**	**Multivariate analysis**	
	***P***	**HR (95% CI)**	***P***	***P***	**HR (95% CI)**	***P***
Age	0.005	1.01 (0.98–1.03)	0.585	0.225		
Gender (male)	0.076			0.207		
BMI	0.036	1.09 (0.99–1.20)	0.083	0.166		
SBP	0.499			0.927		
DBP	0.122			0.065		
Smoking	0.219			0.081		
CVD	< 0.001	2.38 (1.70–5.30)	0.002	0.007	2.04 (1.53–4.67)	0.005
DM	0.004	0.86 (0.39–1.88)	0.699	0.019	2.35 (1.03–5.39)	0.043
Hypertension	0.019	2.21 (0.79–6.20)	0.132	0.185		
Hemoglobin	0.968			0.650		
BUN	0.001	0.95 (0.91–0.99)	0.026	0.012	0.92 (0.86–0.98)	0.013
Creatinine	0.314			0.217		
iPTH	0.028	1.00 (0.99–1.00)	0.109	0.051		
Ca	0.361			0.296		
P	0.076			0.072		
Total *Kt/v*	0.002	1.50 (1.14–1.97)	0.004	0.010	1.74 (0.85–3.58)	0.133
Renal *Kt/v*	0.031	1.26 (1.03–1.52)	0.019	0.022	1.41 (0.94–3.87)	0.192
eGFR	0.149			0.150		
AAPR	0.014	0.57 (0.27–1.18)	0.129	0.049	0.51 (0.22–1.18)	0.113

BMI, body mass index; SBP, systemic blood pressure; DBP, diastolic blood pressure; CVD, cardiovascular disease; DM, diabetes mellitus; BUN, blood urea nitrogen; ALP, alkaline phosphatase; iPTH, intact parathyroid hormone; Ca, calcium; P, phosphorus; eGFR, estimated glomerular filtration rate; AAPR, albumin to ALP ratio.

After PSM, the Kaplan-Meier analysis revealed that patients with a low AAPR (i.e., ≤0.36) also had an increased risk of all-cause mortality compared to patients with AAPR > 0.36 (Figure 2B, *P* < 0.05). In addition, a history of DM (HR 1.87, 95% CI 1.03–3.40, *P* = 0.039), BUN (HR 0.94, 95% CI 0.90–0.98, *P* = 0.002), and lower AAPR (HR 1.96, 95% CI 1.06–3.62, *P* = 0.032) independently predicted OS in PD patients (Table 3). Similarly, the AAPR was not an independent indicator of CVD mortality (HR 0.51, 95% CI 0.22–1.18, *P* = 0.113; Figure 2D and Table 4).

The independent inpact of AAPR on all-cause and CVD mortality was performed by three models. In the unadjusted model, high level of AAPR (AAPR > 0.36) exhibited a negative correlation with increasing risk of all-cause mortality (HR 0.53, 95% CI 0.29–0.97, *P* = 0.041; Table 5). The HR of all-cause mortality in the fully adjusted model was 0.25, which meant that, compared with lower AAPR group (AAPR ≤ 0.36), the risk of death in the higher AAPR group was reduced by 75% (HR 0.25, 95% CI 0.11–0.55, *P* = 0.001; Table 5).

**Table 5:** Multiple Cox regression analysis of AAPR in PD patients.

**Variable**	**Unadjusted model**		**Model I**		**Model II**	
	**HR (95% CI)**	***P***	**HR (95% CI)**	***P***	**HR (95% CI)**	***P***
**All-cause mortality**						
AAPR ≤ 0.36	1.0 (ref)		1.0 (ref)		1.0 (ref)	
AAPR > 0.36	0.53 (0.29–0.97)	0.041	0.50 (0.25–0.99)	0.046	0.25 (0.11–0.55)	0.001
**CVD mortality**						
AAPR ≤ 0.36	1.0 (ref)		1.0 (ref)		1.0 (ref)	
AAPR > 0.36	0.54 (0.24–1.24)	0.148	0.55 (0.22–1.32)	0.187	0.98 (0.93–1.02)	0.312

PD, peritoneal dialysis; HR, Hazard ratio.

Model I: adjusted for age, gender, body mass index, systemic blood pressure, diastolic blood pressure, smoking, cardiovascular disease, diabetes mellitus, and hypertension.

Model II: adjusted for model I covariates and hemoglobin, blood urea nitrogen, intact parathyroid hormone, calcium, phosphorus, total Kt/v, renal Kt/v, and estimated glomerular filtration rate.

### Predictive value of the AAPR for survival

To explore the predictive value of AAPR in patients undergoing PD, the area under the receiver operating characteristic (ROC) curve (AUC) for 5-year mortality was analyzed for these populations. Compared to albumin (AUC = 0.558), ALP (AUC = 0.587), and ALP+albumin (AUC = 0.581), the AAPR (AUC = 0.601) had improved power for predicting all-cause mortality in patients undergoing PD (Figure 1A). Similar results were observed when the predictive values were confirmed by the ROC curve analysis after PSM (Figure 1B).

### New prognostic model for OS

**Figure 3:**
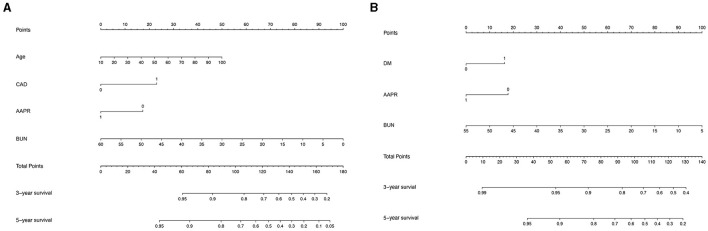
Nomogram built to predict 5-year overall survival in peritoneal dialysis patients before **(A)** and after **(B)** propensity score matching.

**Figure 4:**
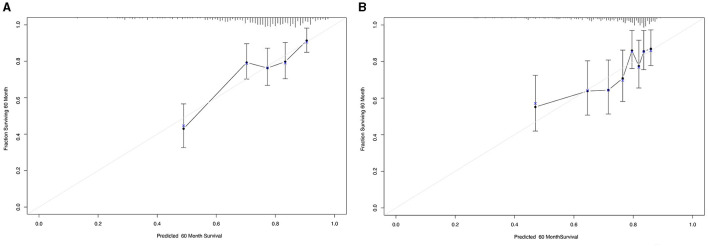
Calibration curves for 5-year overall survival that are representative of the predictive accuracy before **(A)** and after **(B)** propensity score matching analysis. A perfect match between predicted and observed values is represented by the 45-degree reference line.

To further investigate the prediction ability of the AAPR in patients undergoing PD, we built a nomogram based on the results of the multivariate Cox regression model (Figure 3). The respective c-indexes of the nomogram for OS prediction before (Figure 3A) or after (Figure 3B) PAS were 0.68 and 0.71. The performance of the nomograms before (Figure 4A) and after (Figure 4B) in predicting 5-year OS were verified using calibration plots (Figure 4).

## Discussion

Here, we constructed an objective parameter comprised of serum albumin and ALP, the AAPR. We then evaluated its prognostic significance for predicting OS in PD patients. PSM was applied to generate well-balanced groups of patients with low and high AAPR in order to compare survival. The AAPR was identified as an independent indicator of OSin both the entire cohort and the cohort obtained by PSM. We also established a novel nomogram that incorporated AAPR to improve the prediction of mortality in patients undergoing PD.

Links between AAPR and cancer were first introduced for hepatocellular carcinoma patients undergoing curative surgery (13), and subsequent studies showed that, in general, the AAPR can be utilized as a simple indicator in various diseases, such as glioblastoma (14), coronary artery disease (15), non-alcoholic fatty liver disease (16), and renal cell carcinoma (17). In particular, we recently evaluated the AAPR in critically ill patients with acute kidney injury (AKI) and concluded that AAPR independently predicted OS (18). Consistently, the results of the present study confirmed an independent relationship between the AAPR and OS in incident PD patients. In the subgroup analysis, survival outcomes favored a high AAPR across several groups, but not in the group with comorbidities. We found that a low AAPR independently correlated with all-cause mortality in patients undergoing PD, and a cut-off of 0.36 for the AAPR achieved the highest sensitivity and specificity. However, the applicable threshold may be different in further studies due to different sample sizes, follow-up time, and geographic region.

Similar to previous studies (16, 18), the results of this retrospective study suggest that AAPR could be a significant predictor of OS in PD patients and exhibit potential in improving the prediction performance of individual markers alone. As a comprehensive reflection of host's nutritional status, the biological reasons underlying potent prognostic value of AAPR may be elucidated by following reasons. First, as the most abundant serum protein, albumin is conventionally used to evaluate the patient's nutritional status and liver function. However, hypoalbuminemia was recently reported to correlate with poor prognosis in PD patients and was attributed more to inflammation than malnutrition (19). A sufficient amount of albumin is likely to improve intravascular volume and bind inflammatory cytokine, all of which are risk factors for the incidence of PD related peritonitis, resulting in technique failure of PD and increased hophitalization rate and mortality (20, 21). Second, with regard to ALP, it is evidently related to nutritional status and inflammation in CKD (22, 23) and may be further involved in the progression of vascular calcification. Growing evidence has demonstrated an independent relationship between higher ALP levels and adverse clinical outcomes, including hospitalization, cardiovascular events, and mortality in the dialysis population (3, 24). A recent study demonstrated that the alkaline phosphatase to albumin ratio could be a promising non-invasive biomarker for prediciting non-dialysis CKD patients (25). All of these studies led to the conclusion that the AAPR's predictive value is enhanced in PD patients. However, we found that a low AAPR cannot predict cardiovascular outcomes in PD patients, which contradicts our expectation. This may be because bone-specific ALP has a higher specificity and sensitivity than serum ALP in reflecting vascular calcification, which is associated with CVD mortality (26–28). It was recently reported that the effect size related to CVD and non-CVD mortality in dialysis patients was much higher for bone-specific ALP than serum ALP (4). Previous study demonstrated that higher BUN levels were associated with adverse outcomes (29). Conversely, we found that patients with lower BUN levels were at increased risk of mortality, this might be related to restriction of dietary protein intake resulted in a reductction of urea generation (30). Moreover, we found that patients with diabetes on PD were associated with higher all-cause and CVD mortality. Diabetic patients are known at higher risk of hospital admissions, increased rate of infections, peritionitis and mortality (31).

The nomogram utilizes various clinical characteristics and is a generally accepted approach to predicting prognosis (32). In cancers, it is considered to be more precise than the traditional staging systems (33, 34). However, limited evidence is available on the use of nomograms to predict outcomes of PD. In the present study, we developed a prognostic nomogram for 5-year mortality in patients undergoing PD. This nomogram included the AAPR, which was found to be an independent prognostic marker. It performed well for OS even after PSM and was supported by the calibration curve. Therefore, our results revealed that the AAPR could be considered when predicting prognosis in PD patients.

A primary advantage of the current study is the relatively large sample size from multiple centers. We demonstrated good prognostic performance of the AAPR in PD patients using univariate and multivariate models, as well as PSM. The patients' clinical characteristics were well-matched to reduce potential confounding bias, which allows a robust conclusion regarding the independent prognostic significance of the AAPR. However, there are several limitations that should be acknowledged. First, although PD-related variables were widely collected for this study, some variables were not available, such as prior peritonitis episodes and residual kidney function, which may result in inevitable residual confusion. Second, we cannot confirm changes in the AAPR during PD treatment or whether dynamic changes affect the prognosis of PD. Additionally, it is meaningful and advantageous to use a joint modeling framework to estimate time-dependent ROC curve since it evaluates the association between longitudinal markers and the corresponding event-time processes (35). Finally, the mechanisms underlying the AAPR and renal failure biology were not investigated and require further confirmation.

## Conclusion

In conclusion, our study suggests that AAPR could be used as an independent predictor of OS in patients undergoing PD. The AAPR, as well as the newly developed prediction nomogram, may help determine the clinical prognosis of PD and guide individualized treatment.

## Data availability statement

The raw data supporting the conclusions of this article will be made available by the authors, without undue reservation.

## Ethics statement

The study was approved by the Medical Ethics Committee of Jiangyin People's Hospital Affiliated to Nantong University, Affiliated Hospital of Yangzhou University, Yangzhou First People's Hospital, Affiliated Hangzhou First People's Hospital, Zhejiang University School of Medicine and Suzhou Municipal Hospital, it was conducted in accordance with the guidelines and regulations of the Helsinki Declaration. The need for informed consent was waived by the Institutional Review Board of Jiangyin People's Hospital Affiliated to Nantong University (IRB number 2021-0220), Affiliated Hospital of Yangzhou University, Yangzhou First People's Hospital (IRB number #376), Affiliated Hangzhou First People's Hospital, Zhejiang University School of Medicine (IRB number #1780), and Suzhou Municipal Hospital (IRB number 20-1206). The studies were conducted in accordance with the local legislation and institutional requirements. Written informed consent for participation was not required from the participants or the participants' legal guardians/next of kin in accordance with the national legislation and institutional requirements.

## Author contributions

WX: Writing – original draft. XH: Data curation, Writing – original draft. DS: Methodology, Writing – review & editing. XX: Supervision, Writing – review & editing. HH: Writing – review & editing.
